# Virtual 2D mapping of the viral proteome reveals host-specific modality distribution of molecular weight and isoelectric point

**DOI:** 10.1038/s41598-021-00797-3

**Published:** 2021-10-28

**Authors:** Tapan Kumar Mohanta, Awdhesh Kumar Mishra, Yugal Kishore Mohanta, Ahmed Al-Harrasi

**Affiliations:** 1 Department of Biotech and Omics, Natural and Medical Sciences Research Center, University of Nizwa, Nizwa, 616 Oman; 2 Department of Biotechnology, Yeungnam University, Gyeongsan, 48541 South Korea; 3 Department of Applied Biology, School of Biological Sciences, University of Science and Technology Meghalaya, Techno City, Baridua, Ri-Bhoi, Meghalaya 793101 India

**Keywords:** Virology, Microbiology, Biochemistry, Peptides, Proteins, Proteomics

## Abstract

A proteome-wide study of the virus kingdom based on 1.713 million protein sequences from 19,128 virus proteomes was conducted to construct an overall proteome map of the virus kingdom. Viral proteomes encode an average of 386.214 amino acids per protein with the variation in the number of protein-coding sequences being host-specific. The proteomes of viruses of fungi hosts (882.464) encoded the greatest number of amino acids, while the viral proteome of bacterial host (210.912) encoded the smallest number of amino acids. Viral proteomes were found to have a host-specific amino acid composition. Leu (8.556%) was the most abundant and Trp (1.274%) the least abundant amino acid in the collective proteome of viruses. Viruses were found to exhibit a host-dependent molecular weight and isoelectric point of encoded proteins. The isoelectric point (*pI*) of viral proteins was found in the acidic range, having an average *pI* of 6.89. However, the *pI* of viral proteins of algal (*pI* 7.08) and vertebrate (*pI* 7.09) hosts was in the basic range. The virtual 2D map of the viral proteome from different hosts exhibited host-dependent modalities. The virus proteome from algal hosts and archaea exhibited a bimodal distribution of molecular weight and *pI*, while the virus proteome of bacterial host exhibited a trimodal distribution, and the virus proteome of fungal, human, land plants, invertebrate, protozoa, and vertebrate hosts exhibited a unimodal distribution.

## Introduction

Viruses are the most abundant and ubiquitous obligate intracellular parasites^1,2^ with a polyphyletic origin. They also found as virophage where a larger virus act as host for the smaller virus^3,4^. Viruses are sometimes referred to as a “piece of bad news” wrapped in a protein^5^ and lipid^6^. Viruses play a very important role in the ecology and evolution of the biosphere due to their potential ability to integrate into a host genome (except RNA virus and virophage) and utilize the transcription and translation machinery of their host^7,8^. Although most RNA viruses cannot, a small number of viruses (e.g. HIV-1) has the potential that can use reverse transcriptase to produce DNA that can integrate into the host genome^9^. Viruses are obligate parasites whose genomes have played an important role in the evolution of life^10^. Almost all viruses use single-stranded or double-stranded DNA or RNA for genome replication and expression^11,12^. The evolutionary origin of viruses, however, is a mystery due to their patchy molecular and functional attributes^13,14^. Although several theories and hypotheses^15^ have been postulated regarding the origin and evolution of viruses, none are backed by substantial evidence. Phylogenetic and taxonomic studies of viruses have gained enormous attention as the number of sequenced viral genomes has rapidly increased^16^. An understanding of the taxonomy and phylogeny of viruses will help to clarify the diversity and evolution of viruses in different host phyla^16^. Viruses have been extensively used in medicine and for genetic engineering due to their simple genetic architecture^17,18^. In addition, viral genomes have also been widely used to better understand the process of gene and DNA replication, transcription, RNA formation, translation, protein formation, and immunology^19^. Viruses are also used as vectors in genetic engineering applications and also as biological warfare agents. Millions of virus species have been projected to exist with approximately 6000 virus species thus far reported^20^.

The most challenging aspect of deciphering the origin and evolution of viruses is the presence of high sequence divergence due the high rate of mutation, genetic recombination, gene duplication/loss, and horizontal gene transfer that occurs in viruses^21,22^. The high level of sequence divergence and the smaller number of genes in viral genomes relative to prokaryotic and eukaryotic genomes, makes it difficult to identify genes that are conserved across and within families of viruses. The mutation rate of the viral genome varies from 1.5 × 10^–3^ mutation per nucleotide per genomic replication (RNA phage Qβ) to 1.8 × 10^–8^ mutations per nucleotide per replication (HSV-1)^22–24^. Notably, the mutation rate corresponds to the type of polymerase used in replication^22^. RNA viruses that use an RNA-dependent RNA polymerase were reported to mutate faster than viruses that use an RNA-dependent DNA polymerase (retrovirus) or reverse transcriptase^22^. Viruses that use an RNA-dependent RNA polymerase also mutate faster than viruses that use a DNA polymerase^22^. Drake et al. proposed a universal mutation rate in microorganisms of 3.4 × 10^–3^ mutations/genome/genomic replication^25^. The mutation rate of ssDNA (phage ϕX174), however, was reported to be quite higher than the level proposed by Drake et al.^26^. The high mutation rate makes it challenging to address problems associated with viral diseases. Although the genomic aspects of viruses are frequently studies, the proteome of viruses is poorly understood. Viral genomes, their role in immune modulation, and the use of viral genomes in vaccine development have gained enormous attention. Again, however, the viral proteome, its composition and function across hosts is poorly understood.

Proteomics is a very promising approach that can be used to better understand the molecular details of viruses. Increased knowledge of viral proteomes would enable a better understanding of the disease process and could be used to identify new biomarkers for the diagnosis and early detection of diseases and for drug development. Although proteomics has been used for biomarker development, its use in the development of broad-spectrum vaccines remains limited. The diversity of genomic and proteomic sequences in viruses may explain the basis of this problem. Although viral genomes, proteomes, and even the sequence of individual proteins are not conserved, a portion of a protein sequence can be used to develop a broad-spectrum vaccine against viral diseases. Therefore, characterizing the proteomic details of all known viruses was very important. Viruses are host specific and all viruses require a host to propagate. Therefore, the question arises, whether the genomic and proteomic content of a virus is also host specific? When viruses lack either conserved DNA or protein consensus sequences, it becomes essential to analyse viral genomes and proteins as they relate to host specificity. It is plausible that viruses may use similar molecular or biochemical mechanisms to propagate in specific hosts or host ranges. Understanding the molecular details of different groups of viruses in relation to their hosts may shed more light on the evolution and phylogeny of viruses. Therefore, we conducted a proteome-wide analysis of the proteome of the virus kingdom to provide details pertaining to the composition and structure of the proteome of the virus kingdom.

## Results

### Viruses exhibit diverse genomic and proteomic characteristics

To understand the proteomic details of the virus kingdom, authors have downloaded 1.713 million predicted virus protein sequences from 19,128 viruses from National Center for Biotechnology Information database. Result showed, viruses exhibit great diversity in genome size, ranging from the largest at 49.31 Mb (uncultured marine virus, GCA_002003225.1) to the smallest at 0.001043 Mb (GCA_004205475.1), the latter of which comprises a circular virus. A few other viruses with large genome sizes are 31.04 Mb (GCA_002003145.1), 29.61 Mb (GCA_002003185.1), 26.49 Mb (GCA_002013235.1), 25.31 Mb (GCA_002013255.1), 25.05 Mb (GCA_002003165.1), 21.19 Mb (GCA_002003205.1), 16.98 Mb (GCA_002003245.1), and 6.68 Mb (GCA_002003125.1) for uncultured marine viruses of invertebrate hosts. Due to the lack of properly annotated protein coding sequences, these viruses were excluded from the present study. Only viral genomes with complete protein annotation were utilized in the present analysis. Among these, *Hyperionvirus* sp. (GCA_003814165.1), with a genome size of 2.38 Mb, was found to encode the highest number of protein sequences (2493), followed by *Pandoravirus inopinatum* (2.24 Mb, GCA_000928575.1) with 1839 protein coding sequences. The average viral genome size was calculated to be 0.038 Mb and encodes an average of 48.662 protein sequences. Notably, a circular virus (GCA_004205475.1) was found to encode only one protein sequence within its 0.001043 Mb genome sequence.

Viruses were found to encode a variable number of coding sequences, the number of which was based on their host. On average, the virus proteome was found to encode 386.214 amino acids per protein sequence. The average number of amino acids found in protein sequences of the virus proteome varied depending on their host species, as follows: algae (254.68), archaea (216.774), bacteria (210.912), fungi (882.464), humans (388.459), invertebrates (390.741), land plants (405.099), protozoans (329.364), and vertebrates (397.433). The protein sequences of bacterial viruses were found to be the smallest (210.912) and fungal viruses encoded he largest proteins (882.464). PSCNV polyprotein (AYD75846.1) of the Planarian secretory cell nidovirus was the largest and heaviest viral protein, comprising 13,556 amino acids and 1567.858 kDa. The largest viral proteins in other hosts were: fibronectin type III domain-containing protein (8212 amino acids, 876.302 kDa) of algal Tetraselmis virus 1 (accession AUF82581.1), pp1ab protein (8108 amino acids, 925.221 kDa) of the vertebrate Ball python nidovirus 1 (accession AIJ50565.1), hypothetical polyprotein (7391 amino acids, 829.958 kDa) of the land plant Gentian Kobu-sho-associated virus (accession BAM78286.1), baseplate wedge initiator protein (7312 amino acids, 800.148 kDa) of the bacterial *Prochlorococcus* phage P-SSM4 (accession AAX46881.1), ORF1ab (7182 amino acids, 810.941 kDa) of the human SARS coronavirus (accession AAT98578.1), polyprotein (6359 amino acids, 703.133 kDa) of the fungal Ceratobasidium endornavirus D (accession AOV81681.1), hypothetical protein (5699 amino acids, 639.34 kDa) of the archaea Prokaryotic dsDNA virus sp. (accession QDP67171.1), and FhaB-like adhesion protein (4840 amino acids, 497.539 kDa) of the protozoan Bodo saltans virus (accession ATZ80495.1).

### Small peptides encoded in viral genomes

Viruses were also found to encode small peptides. Murine hepatitis virus (vertebrate host) was found to encode the smallest peptide (CAA28656.1) in the known virus kingdom with only 5 amino acids, M-S-S-T-T. A putative NTPase/helicase (AIW68525.1) in common midwife toad virus was found to encode seven amino acids, M-L-I-S-L-S-E. Hypothetical protein (AHZ10898.1) in the protozoan *Leishmania aethiopica* RNA virus was found to encode eight amino acids, M-I-Q-C-S-T-V-A. Hypothetical protein (AAQ13720.1) in the archaea His 1 virus was found to encode 15 amino acids, M-Q-M-Q-E-K-G-W-K-I-I-I-E-E-Q, and hypothetical protein phiC2p81 (ABE99541.1) of the *Clostridium* virus was found to encode a small peptide of 15 amino acids, M-G-I-S-G-E-K-L-G-N-F-L-I-F-K. The fungal *Sclerotinia sclerotiorum* fusarivirus 1 was found to encode a small peptide of 28 amino acids (hypothetical protein, AKJ26311.1).

### Viruses exhibit host-specific amino acid composition

**Figure 1:**
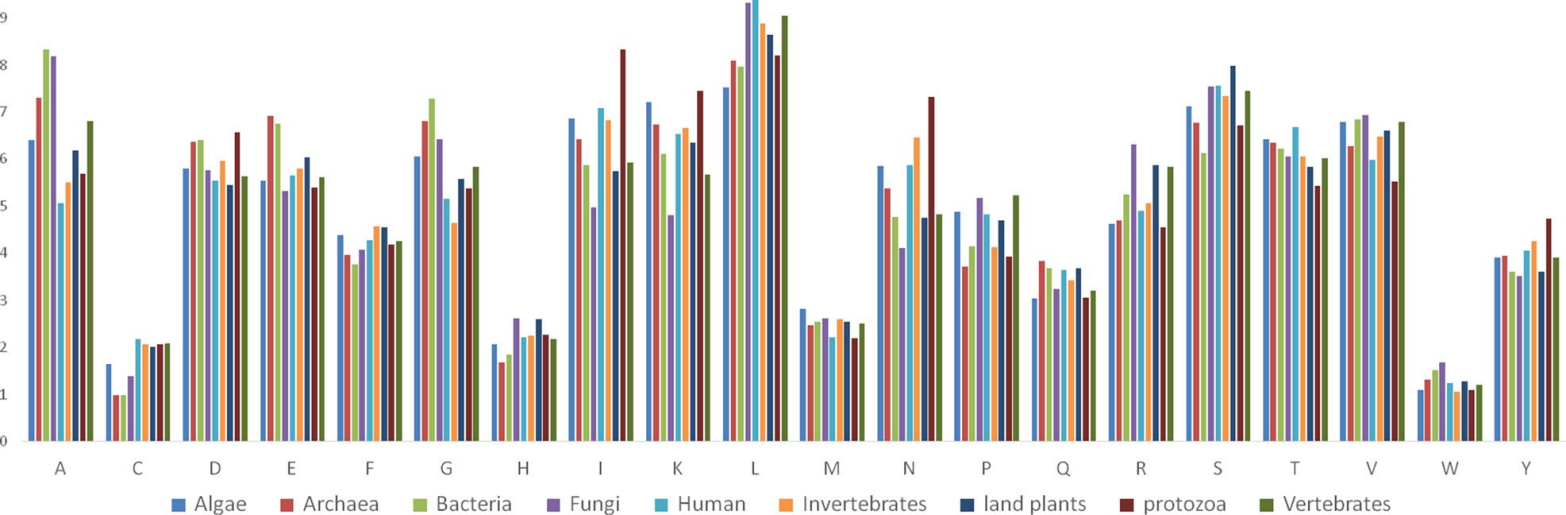
Individual amino acid composition of the virus proteome. Ala (A) amino acid was found to be highest in viruses of bacteria and fungi and lowest in the human host. Similarly, Ile (I) amino acid was found highest in the virus proteome of the protozoan host. The letters in the X-axis represent the initials of 20 essential amino acids. The graph was generated using Microsoft excel version 2016.

An analysis of 1.713 million viral protein sequences from 19,128 virus proteomes across many different hosts was conducted. The viruses were grouped according to their host. The host groups were Algae (99), Archaea (555), Bacteria, (10,116) Fungi (312), Human (540), Invertebrate (1956), Land plants (2546), Protozoa (87), and Vertebrates (2917). The amino acid composition in the different virus proteomes was analysed to characterize host-specific amino acid distribution (Supplementary Table 1). Cys (2.18%), Leu (9.37%), and Thr (6.669%) amino acids were in the highest percentage in human virus proteomes (Fig. 1). Ala (8.331%) and Gly (7.285%) were found in the greatest abundance in bacterial viruses (Fig. 1). The highest percentage of Asp (6.564%), Ile (8.333%), Lys (7.455%), Asn (7.314%), and Tyr (4.723%) was found Protozoan viruses; Glu (6.908%) and Gln (3.827%) were the most abundant in Archaea viruses, while His (2.626%), Arg (6.307%), Val (6.935%), and Trp (1.680%) were most abundant in fungal viruses (Fig. 1, Supplementary Table 1).

Cys (0.983%), His (1.680%), and Pro (3.728%) amino acids were least abundant in Archaea viruses. Glu (5.310%), Ile (4.972%), Lys (4.795%), Asn (4.111%), and Tyr (3.525%) exhibited their lowest abundance in fungal viruses. The lowest percentage of Phe (3.755%) and Ser (6.123%) was found bacterial viruses, Met (2.202%), Arg (4.540%), Thr (5.425%), and Val (5.520%) exhibited their lowest abundance in Protozoan viruses (Fig. 1, Supplementary Table 1).

**Figure 2:**
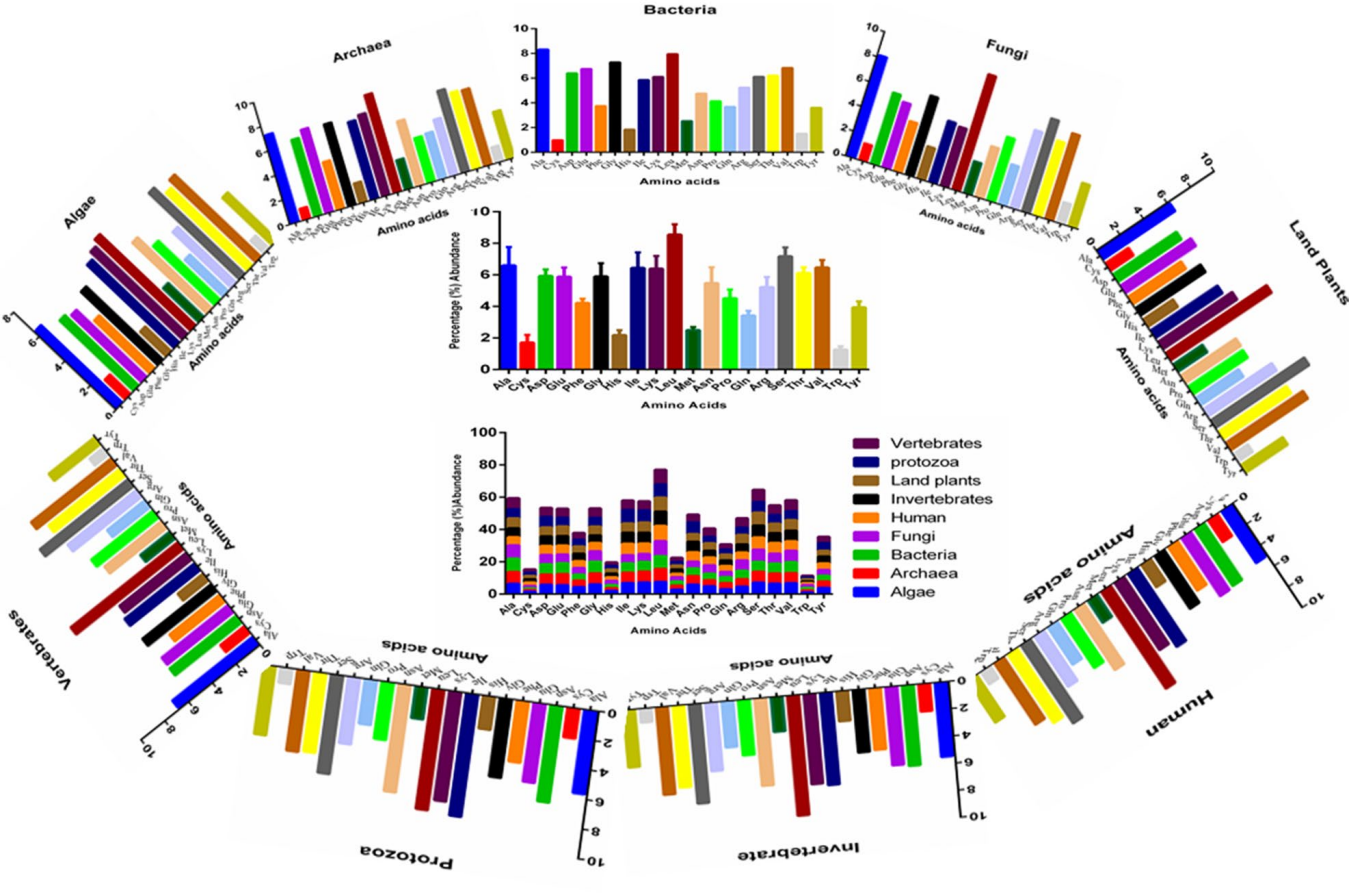
Host-specific amino acid composition of the virus proteome. It was found that Leu (8.556%) was highest and Trp (1.274%) was the lowest abundant amino acid of the virus kingdom. The stacked chart represents the amino acid composition percentage share of virus proteome originated from different hosts. The graphs were generated using GraphPad Prism version 6.

The average amino acid composition of all virus proteomes collectively, indicated that Leu (8.556%) was the most abundant and Trp (1.274%) was the least abundant (Fig. 2, Supplementary Table 1). A few of the other high abundant amino acids were Ser (7.176%), Ala (6.605%), and Val (6.467%) (Fig. 2, Supplementary Table 1). Similarly, a few of the low abundant amino acids in the collective virus proteome were Cys (1.71%), His (2.19%), and Met (2.501%) (Fig. 2). However, selenocysteine and pyrrolysine amino acid was not found in the virus proteome.

A principal component analysis was conducted to determine the relationship (clustering) between the amino acids (Supplementary Figure 1). Results indicated that Gln, Trp, Met, and Thr clustered together, Asn and Ile also clustered together (Supplementary Figure 1). This clustering reflects the presence of the most abundant amino acids in Protozoan viruses and the least abundant amino acids in fungal viruses (Supplementary Figure 1, Supplementary Table 1). The amino acid composition of human and invertebrate viruses clustered together while the composition of land plant and vertebrate viruses also clustered together (Supplementary Figure 1). Bacterial and Archaeal viruses also clustered near each other (Supplementary Figure 1).

**Figure 3:**
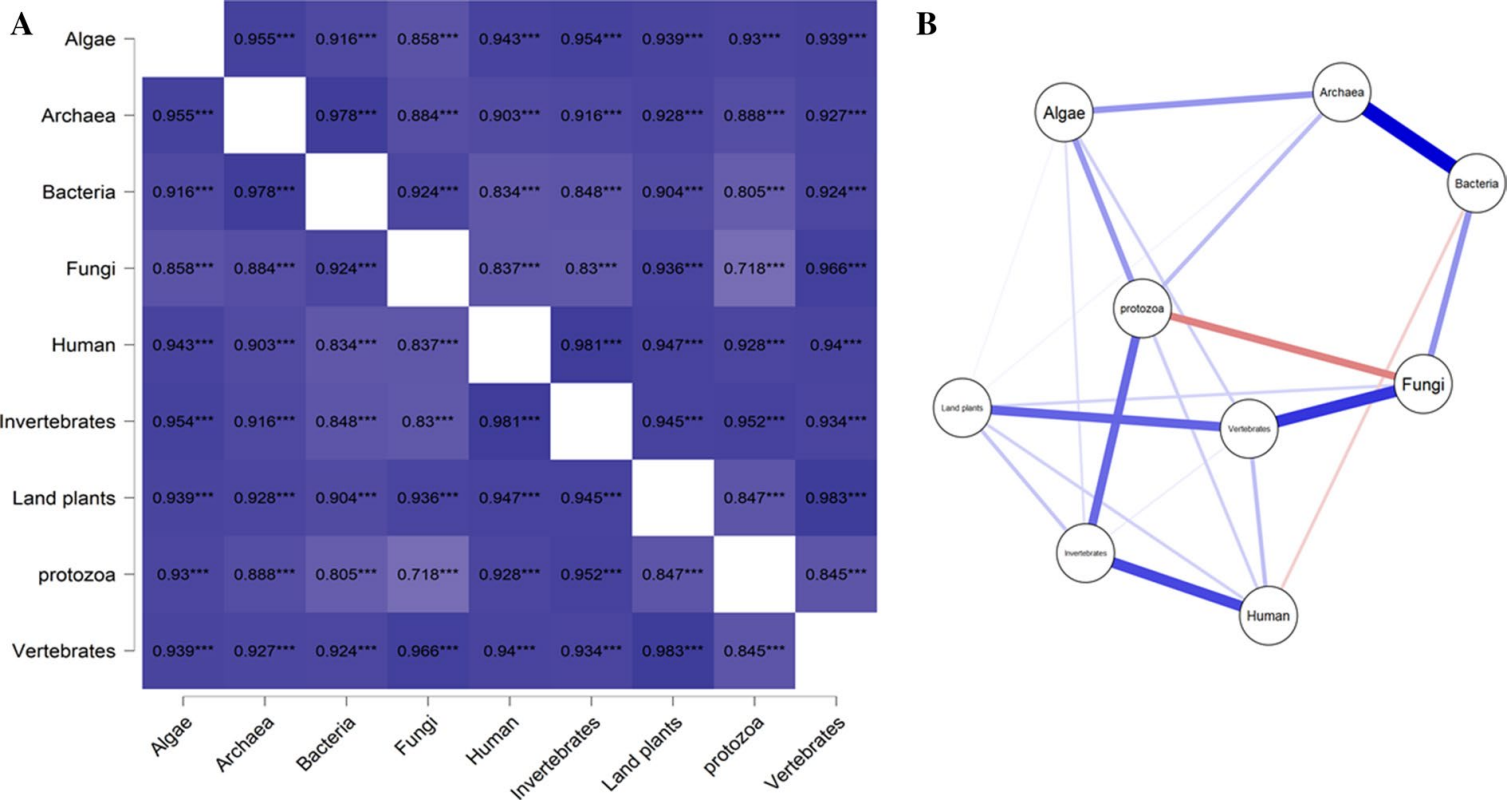
(**A**) Heatmap and (**B**) network plot of the amino acid composition of the virus proteome originated from different hosts. Heatmap shows, maximum of the virus protein shows a positive correlation on its amino acid composition. The highest positive correlation was found between the virus proteome of bacteria and archaea (0.978) whereas the lowest correlation was found between virus proteome originated from fungi and protozoa (0.718). The dark blue mark indicates the highest and the red mark indicates the lowest correlation. Pearson’s correlation study was conducted to construct the correlation plot (p < 0.05).

**Figure 4:**
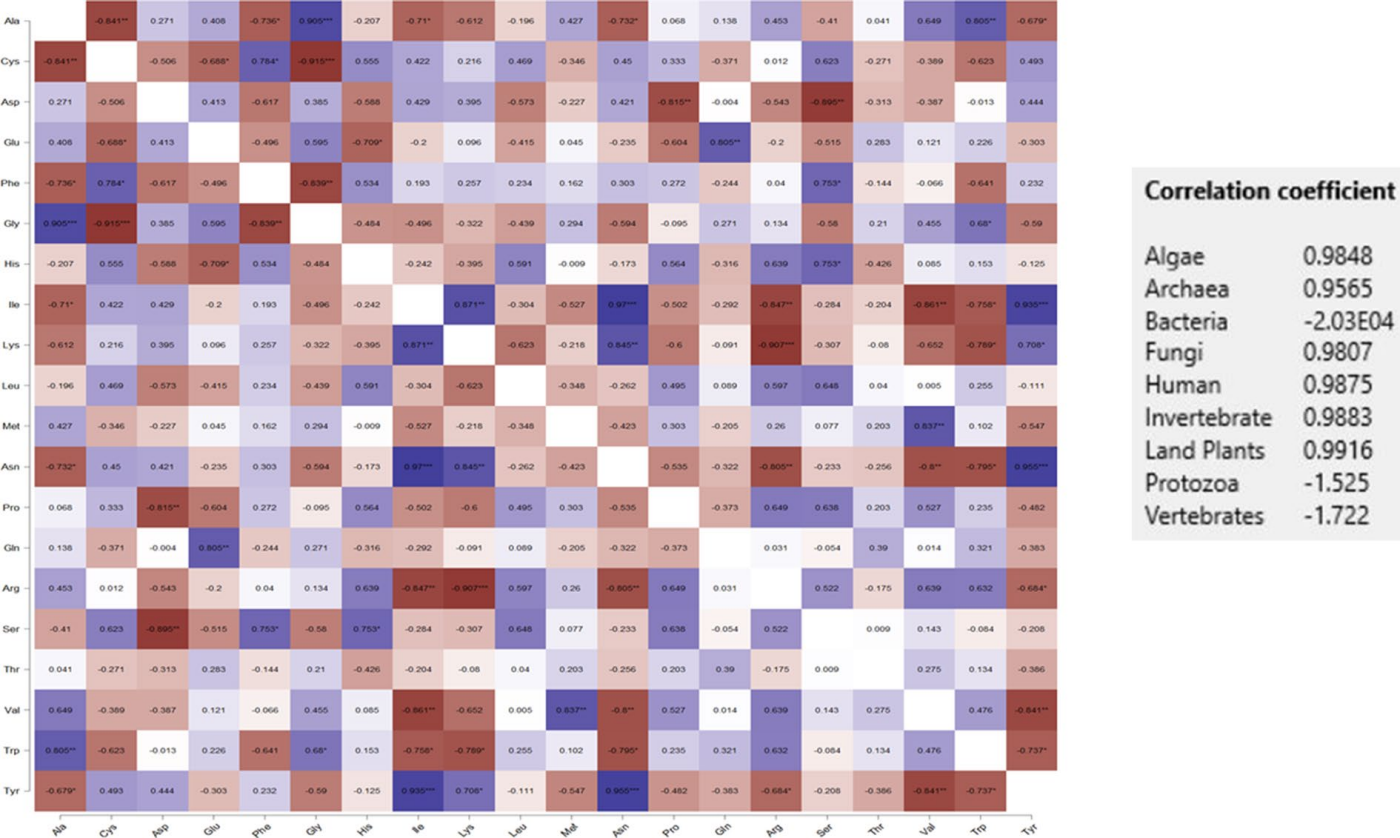
Correlation heat map of the composition of viral amino acid originated from different hosts. The maximum of the amino acids showed a negative correlation (red) whereas only a few amino acids showed a positive correlation (blue). The protein sequences of the virus originated from host land plants showed a positive correlation coefficient (0.9916) whereas protein sequences of the virus originated from bacteria showed a negative correlation (− 2.03E04). Pearson’s correlation study was conducted to construct the correlation plot (p < 0.05).

A correlation analysis was conducted to determine the relationship between amino acid composition in viruses that infect different hosts (Fig. 3). Results revealed that all of the amino acids were positively correlated with the viruses of different hosts. The amino acid composition of in algae with Archaea, Bacteria, Human, Invertebrate, land plant, Protozoa, and Vertebrate hosts exhibited a positive correlation coefficient of > 0.90 (Fig. 3). The correlation between Algae and Fungi; Bacteria and humans and invertebrate, Fungi and humans and invertebrates, Protozoa and Archaea, Bacteria, fungi, land plants, and vertebrates exhibited a positive correlation (Pearson) to a lesser extent. The lowest correlation was observed between Fungi and Protozoa (0.718), while the highest correlation was observed between Archaea and Bacteria (0.978) (Fig. 3). When correlation analysis was done without grouping the amino acids by host species, both positive and negative correlations were observed. Gly and Trp exhibited a positive correlation with Ala, Phe had a positive correlation with Cys, Gln was positively correlated with Glu, Cys and Ser were positively correlated with Phe, Ala was positively correlated with Gly; Lys, and Asn, and Tyr was positively correlated with Ile. Ile, Lys, and Tyr were positively correlated with Asn, Phe and His were positively correlated with Ser, Met was positively correlated with Val, and Ala was positively correlated with Trp (Fig. 4). The highest positive correlation was found between Ile and Asn (0.970), followed by Tyr and Asn (0.955) (Fig. 4). Notably, Cys, Phe, Ile, Lys, Asn, Ser, and Try were negatively correlated with Ala. Ala, Asp, Glu, Gly, Met, Gln, Thr, Val, and Trp were negatively correlated with Cys. Cys, Phe, His, Leu, Met, Pro, Arg, Ser, Thr, and Val were negatively correlated with Asp. Ala, Cys, Asp, Glu, Gly, and Trp were negatively correlated with Phe (Fig. 4). Other negative correlations can be observed as well in Fig. 4. The strongest negative correlation was found between Cys and Gly (− 0.915), followed by Arg with Lys (− 0.907) (Fig. 4). Overall, the correlation analysis indicated, that the amino acids in viruses associated with algae (0.9848), archaea (0.9565), fungi (0.9807), humans (0.9875), invertebrates (0.9883), and land plants (0.9916) exhibited a positive correlation coefficient, while the amino acids of viruses associated with bacteria (− 2.03E04), protozoa (− 0.525), and vertebrates (− 1.722) exhibited a negative correlation (Fig. 4).

### The molecular weight of viral proteins is linked to their viral hosts

Viruses encode proteins with different molecular weights. It was of interest to determine if viruses associated with different hosts contain proteins with a similar or different range of molecular weights. The analysis indicated that bacterial viruses encode the smallest proteins (22.942 kDa), while fungal viruses encode the largest proteins (98.911 kDa) (Supplementary Table 2). The ascending order of the molecular weight of viral proteins in different hosts was 22.942 kDa (bacterial host) < 24.33 kDa (archaea host) < 28.64 kDa (algal host) < 37.493 kDa (protozoan host) < 43.493 kDa (vertebrate host) < 44.121 kDa (human host) < 44.585 kDa (invertebrate host) < 45.922 kDa (land plant host) < 98.911 kDa (fungal host) (Supplementary Table 2).

**Figure 5:**
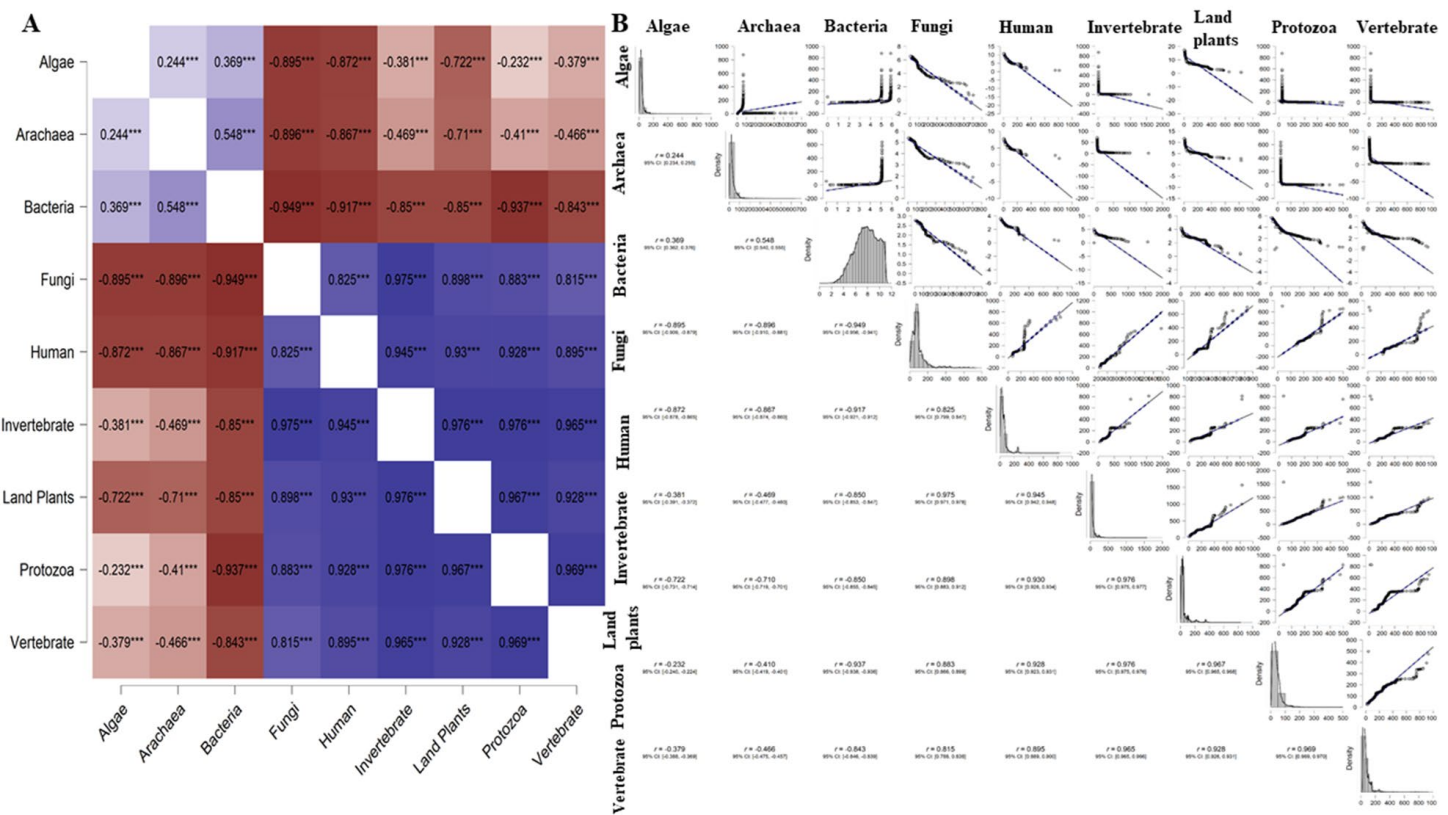
Molecular weight-based correlation analysis of virus proteome. (**A**) Heatmap (**B**) correlation (Pearson’s r) plot. The results show the molecular weight of the virus proteins originated from different hosts. The molecular weight of virus proteins originated from algae, archaea, and bacteria showed a negative correlation with the virus proteins originated from fungi, humans, and land plants; virus proteome of bacteria showed a negative correlation with land plants. However, the virus proteins originated from host fungi showed a positive correlation with virus protein in humans, invertebrates, land, plants, protozoa, and vertebrate; invertebrates with fungi, humans, land plants, protozoa, and vertebrates.

A correlation (Pearson’s) analysis was conducted to determine the relationship between the molecular weight of viral proteins and different hosts (Fig. 5). Results of the analysis indicated that the molecular weight of viral proteins in human, invertebrate, land plants, protozoa, and vertebrate hosts were positively correlated (Fig. 5). The highest correlation was found between invertebrate with protozoan and land plant (0.976) hosts (Fig. 5). A strong positive correlation was also found between invertebrate and vertebrate (0.965) hosts. In contrast, the viral proteins in fungi, human, invertebrate, land plants, protozoa, and vertebrate hosts were negatively correlated with the viral proteins found in algal, archaea, and bacterial hosts (Fig. 5). The strongest negative correlation was between the size of viral proteins in bacterial and fungal hosts (Fig. 5).

#### The pI of viral proteomes is in the acidic range

The *pI* of the virus proteome was found to reside in the acidic *pI* range. The average *pI* of the virus proteome was 6.89. When viral proteomes were grouped by host, however, a host-related *pI* distribution was observed. The bacterial virus proteome exhibited the lowest average *pI* (6.3), while land plant virus proteome had the highest average *pI* (7.5) (Supplementary Table 2). The average *pI* of the algal proteome was *pI* 7.08 and the vertebrate virus proteome *PI* was *pI* 7.09, both of which are in the basic *pI* range. In contrast, the *pI* of the archaeal virus proteome was *pI* 6.42, the *pI* of bacteria viruses was 6.3, fungal viruses (*pI* 6.96), human viruses (*pI* 6.85), invertebrate viruses (*pI* 6.96), and protozoan viruses (*pI* 6.85), all of which represent a basic *pI*.

The decreasing order of the average *pI* of virus proteomes found in different hosts was land plants > vertebrates > algae > fungi > invertebrate > human > protozoa > archaea > bacteria (Supplementary Table 2). The highest *pI* protein in the virus kingdom was a *pI* 13.364 (BAH72951.1) found in the bacterial virus, *Ralstonia* phage phiRSL1, while the lowest *pI* in a virus proteome was *pI* 2.448 (ATZ81137.1), which was found in the protozoan virus Bodo saltans. The average acidic *pI* viral proteome was *pI* 5.507, while acidic *pI* range was 5.001–5.9 (Supplementary Table 3). A principal component analysis (PCA) of acidic *pI* proteins in the entire virus proteome was conducted. The PCA revealed that acidic of *pI* viral proteomes of algae, invertebrates, archaea, vertebrate, and protozoa clustered together. The acidic *pI* viral proteomes of protozoa and vertebrate also clustered together (Supplementary Figure 2). The acidic *pI* viral proteomes of fungi and land plants clustered near each other, while the acidic *pI* of the viral proteomes of humans, and bacteria also clustered in proximity to each other (Supplementary Figure 2). Collectively, the data indicate that acidic *pI* viral proteins exhibit a positive correlation with different hosts.

The average of basic *pI* viral proteomes was *pI* 8.439 and the *pI* range was *pI* 8.13–8.697. A PCA analysis of basic *pI* viral proteomes was conducted (Supplementary Figure 3). PCA analysis indicated that the basic *pI* viral proteomes invertebrates and algae (0.998) clustered together, while the basic *pI* viral proteomes of protozoa and vertebrate clustered in close proximity to each other (Supplementary Figure 3). The basic *pI* viral proteomes of land plants and bacteria clustered close to each other, while those of humans and fungi were far apart (Supplementary Figure 3). The correlation plot of basic *pI* virus protein of different host showed positive correlation from a higher to lower extent. The lowest correlation was found in the case of virus proteins of host bacteria and protozoa (0.892) (Supplementary Figure 3).

### Virtual 2D map of the virus proteome exhibits a host-dependent modality

**Figure 6:**
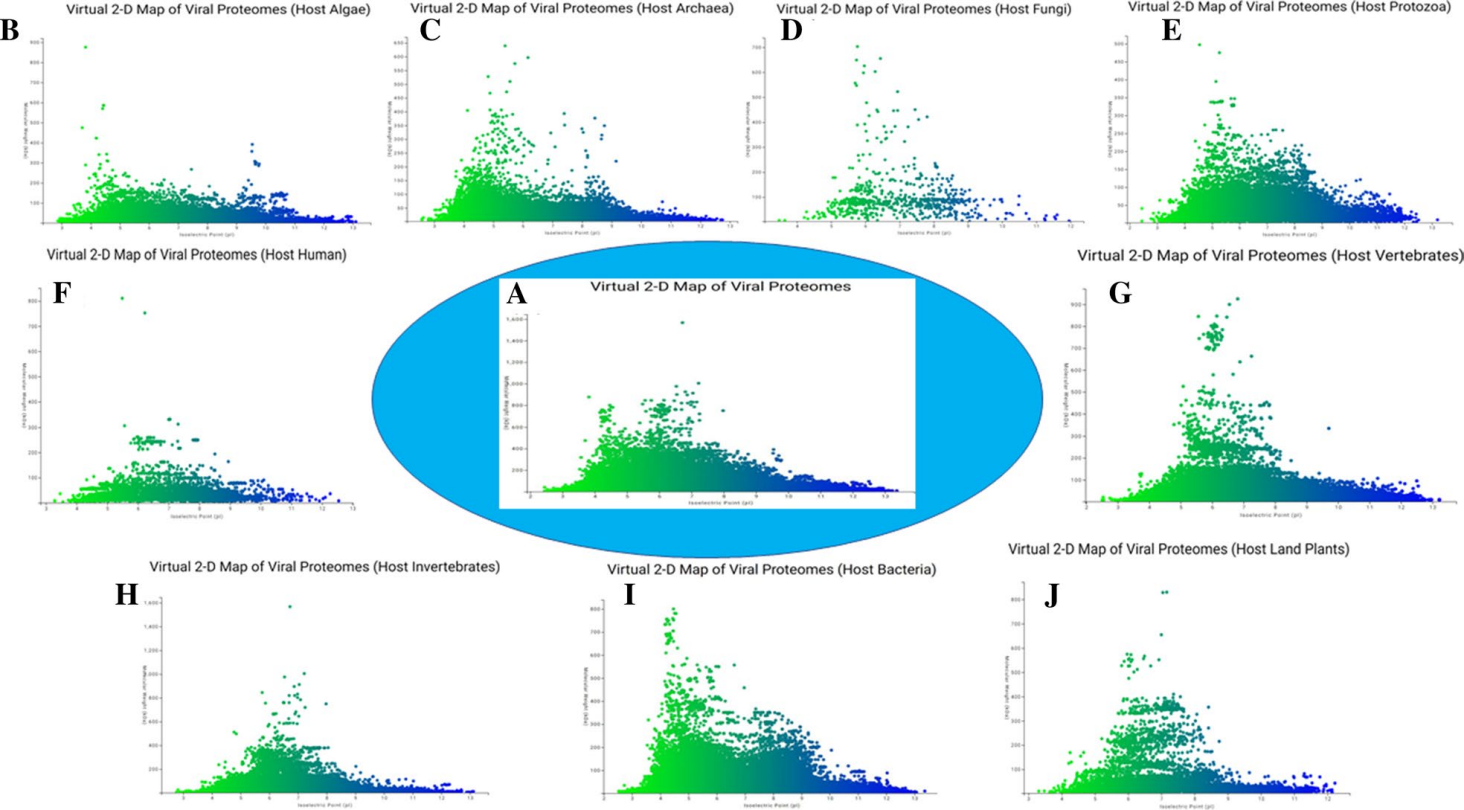
Virtual 2D map of virus proteome. (**A**) The molecular weight and isoelectric point of the virus proteome showed a bimodal distribution. Host-dependent modal distribution of virus proteome can be seen from the figure. Few encode unimodal [(**D**) fungi, (**F**) human, (**H**) invertebrate, (**J**) plants, (**E**) protozoa, and (**G**) vertebrate], a few bimodal [(**B**) algae, (**C**) archaea] and (**I**) bacteria showed trimodal distribution of molecular weight and isoelectric point of virus proteome. The colour opacity represents the acidity and alkalinity of the virus proteome. Green hues tend toward acidity, whereas blue hues tend toward alkalinity. The scatter plots were generated using “scatter plot online” server (https://scatterplot.online/).

A virtual 2D map of the proteome of the virus kingdom was constructed utilizing molecular weight and isoelectric point data (Fig. 6). The virtual 2D proteome map of the virus kingdom exhibits a bimodal distribution in the molecular weight and isoelectric point of viral proteomes (Fig. 6). We subsequently determined if the proteomes within different hosts also exhibit a bimodal distribution. A bimodal distribution was revealed in algal and archaeal virus proteomes, while the viral proteomes of protozoan and bacterial hosts exhibit a trimodal distribution. Lastly, the viral proteomes of fungal, human, land plant, invertebrate, and vertebrate hosts exhibited a unimodal distribution (Fig. 6).

## Discussion

The size of the genome sequences of viruses exhibits a high-level of diversity, ranging from 0.001043 to 49.31 Mb in size. The collective genomes of the virus kingdom encode one to more than 2493 proteins, with an average of 48.662 proteins per genome. The number of amino acid sequences per viral protein in hosts exhibited variation. Fungal virus proteomes encoded proteins with a higher number of amino acids, relative to viral proteomes in other hosts, while the bacterial virus proteome had the smallest number of amino acids per protein. The collective virus proteome was found to encode an average of 386.214 amino acids per protein. The average number of amino acids per protein sequence in fungal and land plant viral proteomes were found to be 882.464 and 405.099, respectively. Notably, the average number of amino acids per protein in the nuclear-encoded (not from chloroplast or mitochondrial encoded) plant proteome was 424.34, while the nuclear-encoded (not from chloroplast) proteome of fungi was 459.319^27,28^. Interestingly, we found that fungal viruses encode larger proteins than the nuclear encoded proteins of its host. This raises the question of why and how this could occur? What are the factors that lead to the production of viral proteins that are larger than those encoded by their fungal hosts? Since viruses do not encode a large number of proteins, perhaps they encode larger proteins that contain a greater number of functional domains, enabling the protein to perform different functions? Viruses were also found to encode small peptides. The smallest peptide of the virus kingdom was revealed as the penta-peptide, M-S-S-T-T, while the smallest peptide encoded in the plant kingdom is a tetra peptide, M-I-M-F, and a dipeptide, M-V, in the fungal kingdom^27,28^. It is interesting to study the functional aspects of small, viral peptides and their molecular activities in host cells have been determined to perform several biological functions^29,30^. These small peptides can regulate hormone levels and may act as hormones or other bioactive agents, such as biocides and anti-cancer drugs^31–35^. Thus, the presence of these small peptides can have enormous impact on their hosts.

The average amino acid composition of virus proteome indicated that Leu (8.556%) is the most abundant and Trp (1.274%) is the least abundant amino acid, respectively. Leu is also the most abundant amino acid in the nuclear-encoded plant (9.62%) and fungal (9.115%) proteome^27,28^. While Trp (1.28%) is the least abundant amino acid in the nuclear-encoded plant kingdom proteome, Cys (1.267%) is the least abundant amino acid in the nuclear-encoded fungal kingdom proteome^27,28^. Cysteine forms disulphide bonds in proteins and provides conformational stability. Disulphide bonds are typically present in extracellular proteins and rarely in intracellular proteins^36^. The low percentage of Cys in viral proteomes reflects their need to be targeted to intracellular compartments of the cell. We suggest that viruses encode a low percentage of Cys amino acids so that their encoded proteins can be readily targeted to the cytoplasm of the host cell. The most and least abundant amino acids in the virus proteome are more closely correlated with their amino acid abundance in the nuclear-encoded proteome of the plant proteome than the fungal proteome. The molecular weight of viral proteomes revealed that the proteomes of fungal viruses encode the heaviest average proteins with an average molecular weight of 98.911 kDa, while the proteomes of bacterial viruses encode proteins with an average weight of only 22.942 kDa. The molecular weight of a protein has a significant impact on translocation across cellular compartments and also represents an important aspect of the functional role of a protein. Viruses are able to manipulate host cells to transcribe and translate large proteins that are not needed by the host cells. Understanding the molecular components responsible for the translation of such large proteins in host cells can be crucial to eliminating virus-mediated diseases in host organisms. The largest protein encoded in the human genome contains more than 27,000 amino acids and possesses at least 34 functional domains, while the largest protein encoded by viruses and produced in human hosts is the ORF1ab polyprotein (AAT98578.1) of the human coronavirus, which contains 7182 amino acids (Supplementary file 1). The largest protein of the virus kingdom is PSCNV polyprotein, which contains 17 putative functional domains (Supplementary file 1). It is plausible that the large number of functional domains in this viral protein reflects the strategy of encoding as many functional domains as possible in a protein so that a stable virus with only a few proteins can exist. Larger protein molecules provide a structural and functional benefit to an organism^37^, a premise that supports viruses encoding a few large proteins with multiple domains rather than many smaller proteins each with a different function.

The average isoelectric point of the virus proteome is in the acidic *pI* range (*pI* 6.89), which is similar to average *pI* of the nuclear-encoded plant and fungal proteomes^27,28^. The average algal virus proteome had a *pI* of 7.08 and the vertebrate viral proteome had a *pI* of 7.09, both of which are in the basic range. Although the collective virus proteome encodes a higher percentage of acidic than basic *pI* proteins, they only encode a few proteins with a neutral *pI* (7.0). The percentage of neutral *pI* viral proteins algal, archaea, bacterial, fungal, human, invertebrate, plant, protozoan, and vertebrate hosts was 0.159%, 0.079%, 0.115%, 0.285%, 0.231%, 0.241%, 0.215%, 0.16%, and 0.241%, respectively. The archaea virus proteomes had the lowest percentage of neutral *pI* proteins, while fungal virus proteomes had the highest percentage of neutral *pI* proteins.

The virus proteome exhibited a host-dependent modal distribution of molecular weight and isoelectric point. Algal and archaea virus proteomes exhibited a bimodal distribution, while protozoan and bacterial virus proteomes exhibited a trimodal distribution. Fungal, human, land plant, invertebrate, and vertebrate viral proteomes exhibited a unimodal distribution. Nuclear-encoded proteomes of the plant kingdom have been reported to exhibit a trimodal distribution, while fungal proteomes exhibit a bimodal distribution^27,28^. Schwartz et al. previously reported a trimodal distribution for the *pI* and molecular weight of all eukaryotic proteins^38^. The pH of cytoplasm of prokaryotic and eukaryotic cells is usually in the acidic *pI* range. Therefore, viruses have also evolved to produce proteins that have an acidic *pI*. However, the pH of chloroplasts is in the basic *pI* range, and as a result, proteins with a basic *pI* may be targeted to specific sub-cellular locations. Kirag et al. reported that the *pI* of a protein is based on their modality and taxonomic association^39^. Ecological niche and sub-cellular localization, however, have also been reported to play a critical role in determining the *pI* of a protein^39^. Schwartz et al. reported that the *pI* of a protein is correlated with its sub-cellular localization and the pH of the cytosol is below 7^38^. Viruses, however, do not have any cellular or sub-cellular components and hence do not encode the *pI* of a protein based on these factors. The selection pressure on the *pI* of a viral protein is completely based on the cellular environment of its host. Modification in the host *pI* environment by external factors can assist in eliminating a virus from its host and decrease the virulence and pathogenicity of a virus. Although the estimated and experimentally-validated *pI* value of a protein can be different in vivo, they are typically in close agreement when evaluated on a 2-DE gel^40^. The variation in the modal distribution of virus proteomes may be attributed to host-dependent selection pressure. The observed variation in the modal distribution of different viral proteomes based on their hosts is intriguing. Protozoan and bacterial virus proteomes exhibited a trimodal distribution, while algal and archaea virus proteomes displayed a bimodal distribution. Although protozoa are eukaryotic organisms and bacteria are prokaryotes, they share a common, trimodal modality in molecular weight and isoelectric point of their proteome. Although, archaea are also prokaryotes, they exhibit a bimodal distribution in the molecular weight and *pI* of their proteomes. The proteomes of multi-cellular eukaryotic hosts, including fungi, humans, land plants, invertebrates, and vertebrates display a unimodal distribution. This perhaps explains why the proteomes of viruses that infect and reside in multicellular eukaryotic hosts also display a unimodal distribution.

## Conclusion

Viruses exhibit highly diverse and heterogeneous genome and replication mechanisms. They tend to undergo a high rate of mutation that contributes to a high degree of genetic and proteomic variation. Similarly, a single-stranded virus mutates more frequently than a double-stranded virus. The reverse transcribing DNA hepadnavirus can undergo a high-degree of genetic mutation that contributes to high genomic and proteomic diversity. In addition, they lack proofreading and lack a DNA repair mechanism. Although, these molecular mechanisms play important role in contributing to higher genetic and proteomic diversity, ecological and demographic factors are also responsible for the higher genetic diversity. Natural selection and random genetic drift generate great pressure to undergo evolution towards the genetic diversity in viruses. Although there is a great diversity between the virus population, the virus population structure for any particular host can be useful to develop broad range of antiviral agents/vaccines using artificial intelligence and a machine learning approach. The virus proteome study is just a starting point for the functional studies and discoveries uncovered through the proteomic study need extensive study to determine their functional significance. Virus proteome-based specific application tool need to be developed to understand valuable information on viral pathogenesis and their lifecycles as well as cellular functions. Amino acid composition, isoelectric point, and molecular weight of the virus protein can be very valuable towards development of such tools in future.

## Materials and methods

### Sequence retrieval and calculation of molecular weight and isoelectric point

All the protein sequences of the virus proteome were downloaded from the National Center of Biotechnology Information (NCBI). The virus proteome was downloaded based on its host. The viral host were algae, archaea, bacteria, fungi, humans, invertebrates, land plants, protozoa, and vertebrates. In total protein sequences of 19,128 virus proteome were downloaded that constituted 1.713 million predicted protein sequences. The downloaded protein sequences of the virus proteome were subjected to analysis of molecular weight and isoelectric point. The molecular weight of the virus proteins was calculated using the python-based command line “protein isoelectric point calculator” (IPC Python) in a Linux-based platform. The source code was used as mentioned by Kozlowski^41^. All the analysis was conducted based on the host groups to understand the host-specific relationship and or differences. The calculated molecular weight and isoelectric point of the virus proteome were further processed using Microsoft excel 2016.

### Statistical analysis

Statistical analysis was conducted to understand the similarity and difference between the virus proteome originated from a different host. Principal component analysis (PCA) is used to analyze exploratory data and for making predictive models by projecting each data points. It defined the direction and maximizes the variance of the projected data. Therefore, to understand the similarity and variances in the virus proteome data we conducted the PCA to understand the similarity and differences of the amino acid composition of the virus proteome. Further, PCA was also used to understand the similarity and variances in the acidic and basic *pI* proteins of the virus proteomes. Statistical software Unscrambler version 10.4 was used to conduct the principal component analysis. NIPLAS (nonlinear iterative partial least square) model was used to conduct the PCA plot with 100 iterations. The role of calibrated to validated residual variance was 0.5 and the ratio of validated to calibrated residual variance was 0.75. Correlation and regression analysis was conducted using statistical software JAPS version 0.14.1.0. Pearson’s correlation (r) was used to run the correlation-regression plot with confidence interval 95% (p < 0.05) and prediction interval 95% (p < 0.05). The correlation heat-map plot was constructed using JAPS version 0.14.1.0 software using Pearson’s correlation (r) with confidence and prediction interval 95% (p < 0.05). The photograph of the virtual 2D proteome map of virus proteome was constructed using scatterplot online platform (https://scatterplot.online/).

## Supplementary Information

Supplementary Information 1.

Supplementary Information 2.

Supplementary Figure 1.

Supplementary Figure 2.

Supplementary Figure 3.

Supplementary Table 1.

Supplementary Table 2.

Supplementary Table 3.

## Data Availability

All the data used in this study was taken from publicly available NCBI database and accession number of each of the data are provided as supplementary file.
