# Supplementary material for: Virtual 2D mapping of the viral proteome reveals host-specific modality distribution of molecular weight and isoelectric point
Source: Sci Rep. 2021 Oct 28;11:21291. doi: 10.1038/s41598-021-00797-3 (PMC8553790; doi:10.1038/s41598-021-00797-3)
Supplement: Supplementary file 1 — Supplementary Information 1. [file 41598_2021_797_MOESM1_ESM.docx]

**Supplementary data 1**

Putative functional domains of Chimeric domains of ORF1ab polyprotein of human SARS corona virus. It was found to be the largest protein of the virus kingdom originated from the human host.

1. Cov_Nsp3A
2. Peptidase_c16
3. Macro
4. Bcov_Nsp3C_C
5. Cov_Nsp3D_UBL
6. Bcov_Nsp3E_NAB
7. Cov_Nsp4C
8. M_Pro
9. Cov_Nsp7
10. Cov_Nsp8
11. Cov_Nsp9_ssRNA_BD
12. Cov_Exon_Mtase_CoACT
13. NIRAN
14. CoV_Nsp12_RDRP (RNA dependent RNA polymerase)
15. Cv_ZBD (zinc binding domain)
16. PSRV_helicase
17. NIV_Exon (Nidovirus 3'-5' exoribonuclease (ExoN) domain profile)
18. Cov_N7_Mtase (Coronavirus (CoV) guanine-N7-methyltransferase (N7-MTase) domain)
19. NIV_2_O_Mtase (Nidovirus 2'-O-methyltransferase (2'-O-MTase) domain)
20. Asp_rich (Aspartic acid-rich region)
21. Phe_rich (Phenylalanine-rich region)
22. Cys_rich (Cys-rich region)
23. ATPase_A (ATP synthase A subunit signature)
24. PKC_PHOSPHO_SITE (Protein kinase C phosphorylation site)
25. MYRISTYL (N-myristoylation site)
26. CK2_PHOSPHO_SITE Casein kinase II phosphorylation site
27. Asn_GLYCOSYLATION N-glycosylation site
28. Tyr_PHOSPHO_SITE_1 Tyrosine kinase phosphorylation site 1
29. TYR_PHOSPHO_SITE_2 Tyrosine kinase phosphorylation site 2
30. RGD Cell attachment sequence
31. AMIDATION Amidation site
32. CAMP_PHOSPHO_SITE cAMP- and cGMP-dependent protein kinase phosphorylation site
33. LEUCINE_ZIPPER Leucine zipper pattern
34. ATP_GTP_A ATP/GTP-binding site motif A (P-loop)

***(Invertebrate host) Chimeric Functional domains of PSCNV polyprotein (AYD75846.1)***

1. ANK_REP_REGION Ankyrin repeat region circular profile
2. ILE_RICH Isoleucine-rich region profile
3. TYR_RICH Tyrosine-rich region profile
4. THR_RICH Threonine-rich region profile
5. ASN_RICH Asparagine-rich region profile
6. PA2_HIS Phospholipase A2 histidine active site
7. ASN_GLYCOSYLATION N-glycosylation site
8. MYRISTYL N-myristoylation site
9. CK2_PHOSPHO_SITE Casein kinase II phosphorylation site
10. PKC_PHOSPHO_SITE Protein kinase C phosphorylation site
11. TYR_PHOSPHO_SITE_1 Tyrosine kinase phosphorylation site 1
12. TYR_PHOSPHO_SITE_2 Tyrosine kinase phosphorylation site 2
13. CAMP_PHOSPHO_SITE cAMP- and cGMP-dependent protein kinase phosphorylation site
14. AMIDATION Amidation site
15. RGD Cell attachment sequence
16. LEUCINE_ZIPPER Leucine zipper pattern
17. ATP_GTP_A ATP/GTP-binding site motif A (P-loop)

***(Algae host) Fibronectin type III domain-containing protein [AUF82581.1, Tetraselmis virus 1]***

1. FN3 Fibronectin type-III domain profile
2. FA58C_3 Coagulation factors 5/8 type C domain (FA58C) profile
3. Ser_rich Serine-rich region profile
4. Thr_rich Thr-rich region profile
5. MYRISTYL N-myristoylation site
6. ASN_GLYCOSYLATION N-glycosylation site
7. PKC_PHOSPHO_SITE Protein kinase C phosphorylation site
8. CK2_PHOSPHO_SITE Casein kinase II phosphorylation site
9. AMIDATION Amidation site

***(Archaea host) hypothetical protein (QDP67171.1)***

1. NLS_BP Bipartite nuclear localization signal profile
2. GH18_1 Glycosyl hydrolases family 18 (GH18) active site signature
3. ALDEHYDE_DEHYDR_GLU Aldehyde dehydrogenases glutamic acid active site
4. ZINC_PROTEASE Neutral zinc metallopeptidases, zinc-binding region signature
5. PKC_PHOSPHO_SITE Protein kinase C phosphorylation site
6. MYRISTYL N-myristoylation site
7. AMIDATION Amidation site
8. CAMP_PHOSPHO_SITE cAMP- and cGMP-dependent protein kinase phosphorylation site
9. CK2_PHOSPHO_SITE Casein kinase II phosphorylation site
10. ASN_GLYCOSYLATION N-glycosylation site
11. TYR_PHOSPHO_SITE_1 Tyrosine kinase phosphorylation site 1
12. RGD Cell attachment sequence
13. TYR_PHOSPHO_SITE_2 Tyrosine kinase phosphorylation site 2

***(Bacteria host) Putative structural protein [AJK27526.1 , Cyanophage P-TIM40]***

1. WD_REPEATS_1 Trp-Asp (WD) repeats signature
2. ASN_GLYCOSYLATION N-glycosylation site
3. PKC_PHOSPHO_SITE Protein kinase C phosphorylation site
4. MYRISTYL N-myristoylation site
5. TYR_PHOSPHO_SITE_2 Tyrosine kinase phosphorylation site 2
6. CK2_PHOSPHO_SITE Casein kinase II phosphorylation site
7. AMIDATION Amidation site
8. CAMP_PHOSPHO_SITE cAMP- and cGMP-dependent protein kinase phosphorylation site
9. TYR_PHOSPHO_SITE_1 Tyrosine kinase phosphorylation site 1
10. RGD Cell attachment sequence

***(Fungi host) Polyprotein (AOV81681.1)***

1. ALPHAVIRUS_MT Alphavirus-like methyltransferase (MT) domain profile
2. RDRP_SSRNA_POS RdRp of positive ssRNA viruses catalytic domain profile
3. CYS_RICH Cysteine-rich region profile
4. ASP_RICH Aspartic acid-rich region profile
5. MYRISTYL N-myristoylation site
6. PKC_PHOSPHO_SITE Protein kinase C phosphorylation site
7. CK2_PHOSPHO_SITE Casein kinase II phosphorylation site
8. ASN_GLYCOSYLATION N-glycosylation site
9. CAMP_PHOSPHO_SITE cAMP- and cGMP-dependent protein kinase phosphorylation site
10. AMIDATION Amidation site
11. TYR_PHOSPHO_SITE_1 Tyrosine kinase phosphorylation site 1
12. RGD Cell attachment sequence
13. ATP_GTP_A ATP/GTP-binding site motif A (P-loop)

***(Land plant host) hypothetical polyprotein (BAM78286.1)***

1. HELICASE_ATP_BIND_1 Superfamilies 1 and 2 helicase ATP-binding type-1 domain profile
2. HELICASE_CTER Superfamilies 1 and 2 helicase C-terminal domain profile
3. IG_LIKE Ig-like domain profile
4. RDRP_SSRNA_POS RdRp of positive ssRNA viruses catalytic domain profile
5. NLS_BP Bipartite nuclear localization signal profile
6. ALA_RICH Alanine-rich region profile
7. PKC_PHOSPHO_SITE Protein kinase C phosphorylation site
8. CAMP_PHOSPHO_SITE cAMP- and cGMP-dependent protein kinase phosphorylation site
9. CK2_PHOSPHO_SITE Casein kinase II phosphorylation site
10. MYRISTYL N-myristoylation site
11. AMIDATION Amidation site
12. ASN_GLYCOSYLATION N-glycosylation site
13. TYR_PHOSPHO_SITE_1 Tyrosine kinase phosphorylation site 1
14. TYR_PHOSPHO_SITE_2 Tyrosine kinase phosphorylation site 2
15. RGD Cell attachment sequence
16. ATP_GTP_A ATP/GTP-binding site motif A (P-loop)

***(Host protozoa) FhaB-like adhesion protein [ATZ80495.1, Bodo saltans virus]***

1. ICA Intramolecular chaperone auto-processing (ICA) domain profile
2. ASN_RICH Asparagine-rich region profile
3. NLS_BP Bipartite nuclear localization signal profile
4. PKC_PHOSPHO_SITE Protein kinase C phosphorylation site
5. MYRISTYL N-myristoylation site
6. ASN_GLYCOSYLATION N-glycosylation site

***(Host vertebrate) pp1ab [AIJ50565.1, Ball python nidovirus 1]***

1. ZF_C3H1 Zinc finger C3H1-type profile
2. NIRAN Nidovirus RdRp-associated nucleotidyl transferase (NiRAN) domain profile
3. RDRP_SSRNA_POS RdRp of positive ssRNA viruses catalytic domain profile
4. CV_ZBD Coronaviridae zinc-binding (CV ZBD) domain profile
5. NIV_EXON Nidovirus 3'-5' exoribonuclease (ExoN) domain profile
6. NIV_2_O_MTASE Nidovirus 2'-O-methyltransferase (2'-O-MTase) domain profile
7. GLN_RICH Glutamine-rich region profile
8. MET_RICH Methionine-rich region profile
9. SER_RICH Serine-rich region profileGLU_RICH
10. Glutamic acid-rich region profile
11. LYS_RICH Lysine-rich region profile
12. CYS_RICH Cysteine-rich region profile
13. SBP_BACTERIAL_1 Bacterial extracellular solute-binding proteins, family 1 signature
14. TRYPSIN_SER Serine proteases, trypsin family, serine active site
15. KAZAL_1 Kazal serine protease inhibitors family signature
16. PKC_PHOSPHO_SITE
17. CAMP_PHOSPHO_SITE cAMP- and cGMP-dependent protein kinase phosphorylation site
18. CK2_PHOSPHO_SITE Casein kinase II phosphorylation site
19. ASN_GLYCOSYLATION N-glycosylation site
20. AMIDATION Amidation site
21. MYRISTYL N-myristoylation site
22. TYR_PHOSPHO_SITE_1 Tyrosine kinase phosphorylation site 1
23. ATP_GTP_A ATP/GTP-binding site motif A (P-loop)
