# Supplementary material for: Virtual 2D mapping of the viral proteome reveals host-specific modality distribution of molecular weight and isoelectric point
Source: Sci Rep. 2021 Oct 28;11:21291. doi: 10.1038/s41598-021-00797-3 (PMC8553790; doi:10.1038/s41598-021-00797-3)
Supplement: Supplementary file 3 — Supplementary Figure 1. [file 41598_2021_797_MOESM3_ESM.pptx]

## Slide 1
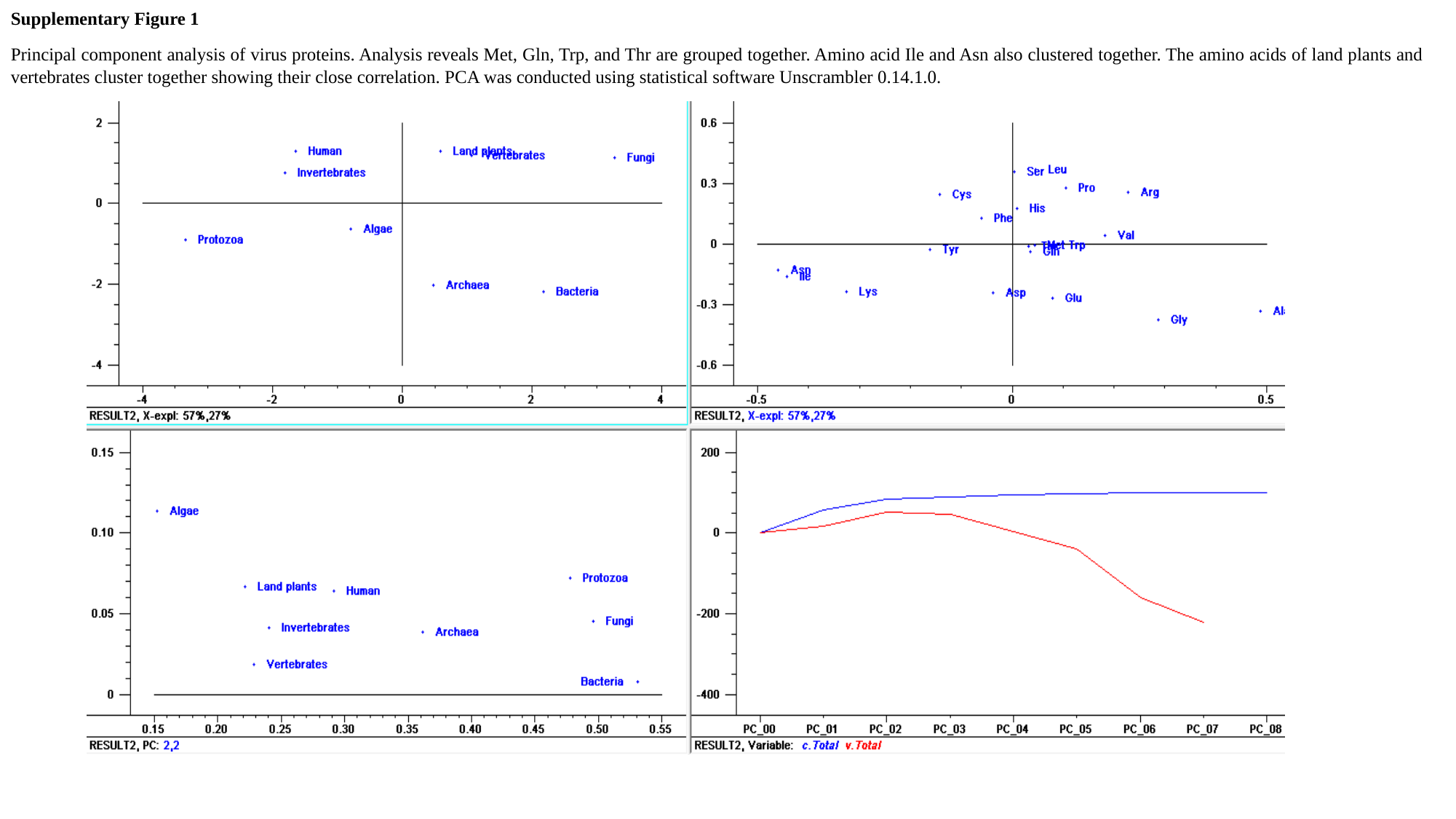

Supplementary Figure 1
Principal component analysis of virus proteins. Analysis reveals Met, Gln, Trp, and Thr are grouped together. Amino acid Ile and Asn also clustered together. The amino acids of land plants and vertebrates cluster together showing their close correlation. PCA was conducted using statistical software Unscrambler 0.14.1.0.
