# Supplementary material for: Virtual 2D mapping of the viral proteome reveals host-specific modality distribution of molecular weight and isoelectric point
Source: Sci Rep. 2021 Oct 28;11:21291. doi: 10.1038/s41598-021-00797-3 (PMC8553790; doi:10.1038/s41598-021-00797-3)
Supplement: Supplementary file 4 — Supplementary Figure 2. [file 41598_2021_797_MOESM4_ESM.pptx]

## Slide 1
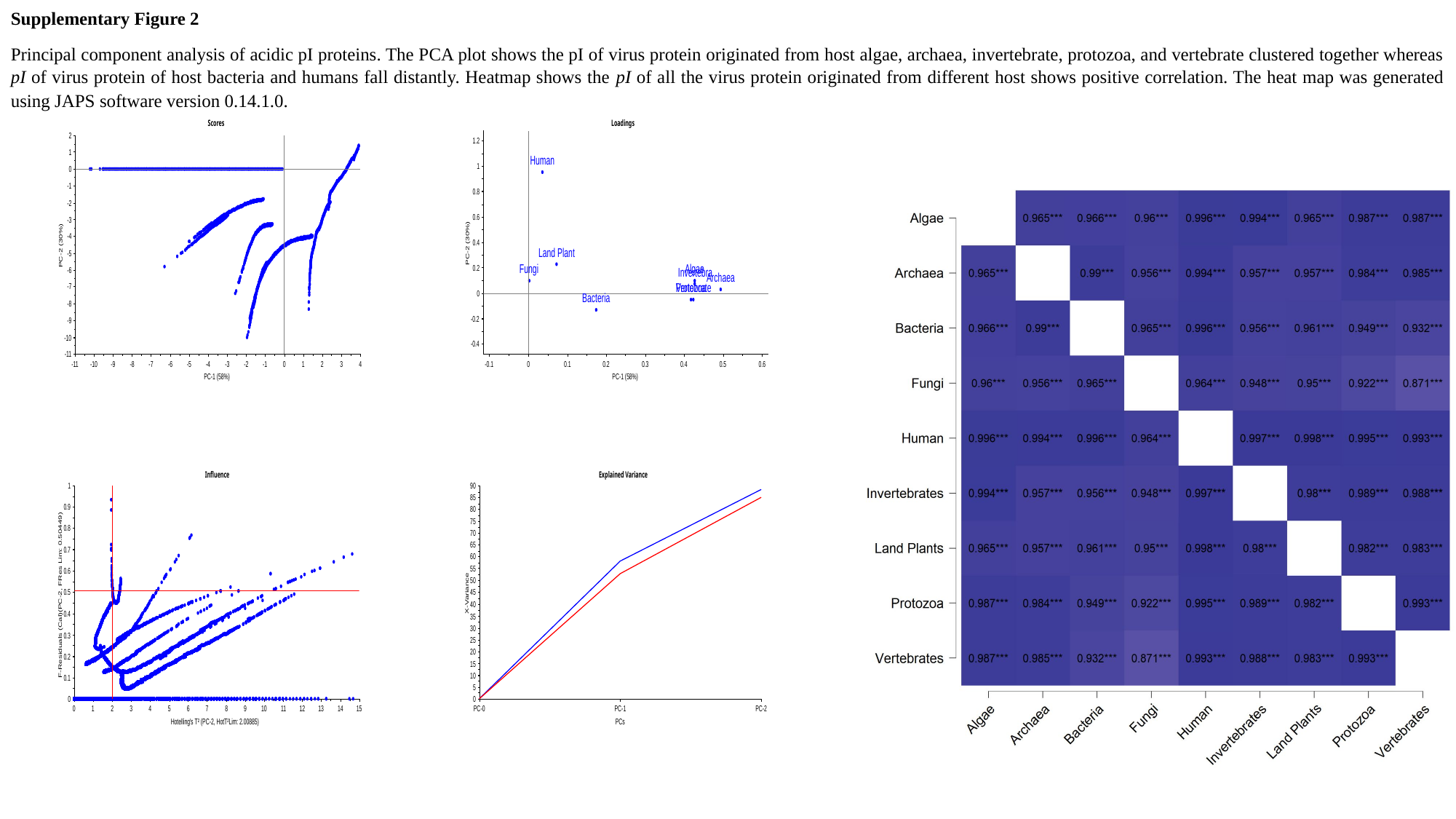

Supplementary Figure 2
Principal component analysis of acidic pI proteins. The PCA plot shows the pI of virus protein originated from host algae, archaea, invertebrate, protozoa, and vertebrate clustered together whereas pI of virus protein of host bacteria and humans fall distantly. Heatmap shows the pI of all the virus protein originated from different host shows positive correlation. The heat map was generated using JAPS software version 0.14.1.0.
