# Supplementary material for: Virtual 2D mapping of the viral proteome reveals host-specific modality distribution of molecular weight and isoelectric point
Source: Sci Rep. 2021 Oct 28;11:21291. doi: 10.1038/s41598-021-00797-3 (PMC8553790; doi:10.1038/s41598-021-00797-3)
Supplement: Supplementary file 5 — Supplementary Figure 3. [file 41598_2021_797_MOESM5_ESM.pptx]

## Slide 1
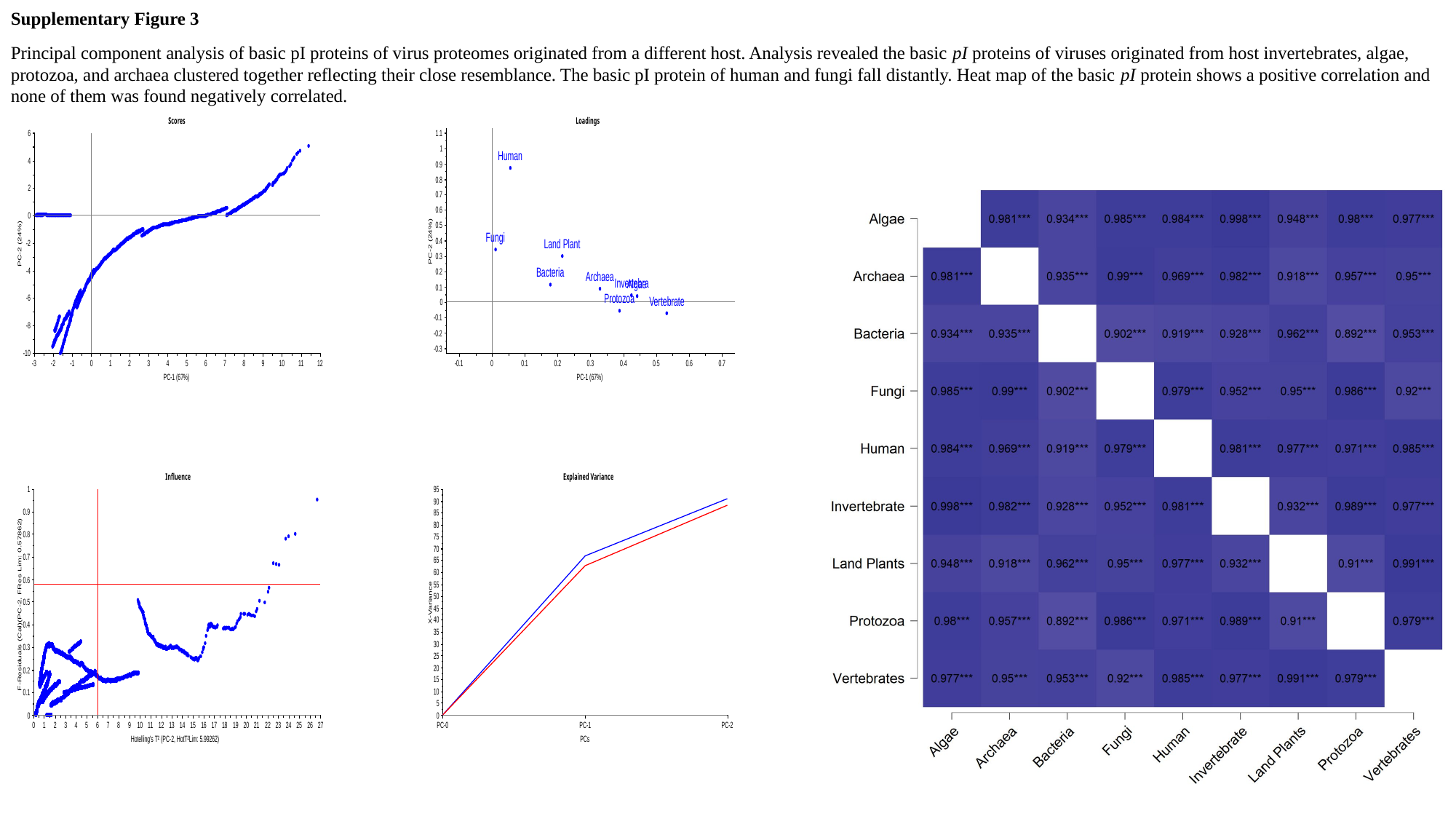

Supplementary Figure 3
Principal component analysis of basic pI proteins of virus proteomes originated from a different host. Analysis revealed the basic pI proteins of viruses originated from host invertebrates, algae, protozoa, and archaea clustered together reflecting their close resemblance. The basic pI protein of human and fungi fall distantly. Heat map of the basic pI protein shows a positive correlation and none of them was found negatively correlated.
