# Supplementary material for: Virtual 2D mapping of the viral proteome reveals host-specific modality distribution of molecular weight and isoelectric point
Source: Sci Rep. 2021 Oct 28;11:21291. doi: 10.1038/s41598-021-00797-3 (PMC8553790; doi:10.1038/s41598-021-00797-3)
Supplement: Supplementary file 6 — Supplementary Table 1. [file 41598_2021_797_MOESM6_ESM.docx]

**Supplementary Table 1**

|  | **Algae** | **Archaea** | **Bacteria** | **Fungi** | **Human** | **Invertebrates** | **Land plants** | **Protozoa** | **Vertebrates** | **Average** | **Highest** | **Lowest** |
| --- | --- | --- | --- | --- | --- | --- | --- | --- | --- | --- | --- | --- |
| Ala | 6.396 | 7.305 | 8.331 | 8.175 | 5.053 | 5.502 | 6.189 | 5.678 | 6.814 | 6.605 | Bacteria | Human |
| Arg | 4.62 | 4.687 | 5.246 | 6.307 | 4.9 | 5.066 | 5.873 | 4.54 | 5.83 | 5.23 | Fungi | Protozoa |
| Asn | 5.857 | 5.375 | 4.762 | 4.111 | 5.868 | 6.461 | 4.747 | 7.314 | 4.827 | 5.48 | Protozoa | Fungi |
| Asp | 5.803 | 6.356 | 6.407 | 5.76 | 5.531 | 5.955 | 5.449 | 6.564 | 5.638 | 5.94 | Protozoa | Land plants |
| Cys | 1.642 | 0.983 | 0.99 | 1.382 | 2.18 | 2.062 | 2.009 | 2.063 | 2.082 | 1.71 | Human | Archaea |
| Gln | 3.04 | 3.827 | 3.689 | 3.249 | 3.645 | 3.422 | 3.691 | 3.065 | 3.205 | 3.426 | Archaea | Algae |
| Glu | 5.536 | 6.908 | 6.75 | 5.31 | 5.649 | 5.804 | 6.036 | 5.388 | 5.603 | 5.887 | Archaea | Fungi |
| Gly | 6.047 | 6.797 | 7.285 | 6.413 | 5.147 | 4.645 | 5.57 | 5.366 | 5.832 | 5.9 | Bacteria | Invertebrates |
| His | 2.061 | 1.68 | 1.846 | 2.626 | 2.221 | 2.243 | 2.597 | 2.267 | 2.172 | 2.19 | Fungi | Archaea |
| Ile | 6.851 | 6.428 | 5.861 | 4.972 | 7.076 | 6.826 | 5.734 | 8.333 | 5.925 | 6.445 | Protozoa | Fungi |
| Leu | 7.524 | 8.084 | 7.956 | 9.313 | 9.373 | 8.874 | 8.633 | 8.193 | 9.049 | 8.556 | Human | Algae |
| Lys | 7.218 | 6.735 | 6.113 | 4.795 | 6.531 | 6.665 | 6.35 | 7.455 | 5.659 | 6.391 | Protozoa | Fungi |
| Met | 2.825 | 2.473 | 2.541 | 2.612 | 2.207 | 2.596 | 2.539 | 2.202 | 2.515 | 2.501 | Algae | Protozoa |
| Phe | 4.384 | 3.968 | 3.755 | 4.063 | 4.272 | 4.561 | 4.55 | 4.176 | 4.26 | 4.221 | Invertebrates | Bacteria |
| Pro | 4.869 | 3.728 | 4.145 | 5.163 | 4.82 | 4.115 | 4.691 | 3.92 | 5.218 | 4.519 | Vertebrates | Archaea |
| Ser | 7.114 | 6.77 | 6.123 | 7.541 | 7.558 | 7.341 | 7.988 | 6.705 | 7.447 | 7.176 | Land plants | Bacteria |
| Thr | 6.419 | 6.353 | 6.225 | 6.051 | 6.669 | 6.057 | 5.83 | 5.425 | 6.019 | 6.117 | Human | Protozoa |
| Trp | 1.086 | 1.314 | 1.512 | 1.68 | 1.247 | 1.051 | 1.286 | 1.092 | 1.198 | 1.274 | Fungi | Invertebrates |
| Tyr | 3.911 | 3.936 | 3.612 | 3.525 | 4.058 | 4.256 | 3.613 | 4.723 | 3.905 | 3.949 | Protozoa | Fungi |
| Val | 6.784 | 6.276 | 6.837 | 6.935 | 5.975 | 6.48 | 6.607 | 5.52 | 6.789 | 6.467 | Fungi | Protozoa |

Amino acid composition (%) of viral proteomes with respect to their host of origin. The highest and lowest amino acid abundance of virus proteome found in different host are also mentioned in the table.
