# Supplementary material for: Virtual 2D mapping of the viral proteome reveals host-specific modality distribution of molecular weight and isoelectric point
Source: Sci Rep. 2021 Oct 28;11:21291. doi: 10.1038/s41598-021-00797-3 (PMC8553790; doi:10.1038/s41598-021-00797-3)
Supplement: Supplementary file 7 — Supplementary Table 2. [file 41598_2021_797_MOESM7_ESM.docx]

**Supplementary Table 2**

| **Host** | **Average of Mol. Weight Viral Protein (kDa)** | **Highest Mol. Weight of Protein (kDa)** | **Lowest Mol. Weight of Protein** | **Highest *pI* of Protein** | **Lowest *pI* of Protein** | **Average *pI* of Viral Proteome** |
| --- | --- | --- | --- | --- | --- | --- |
| Algae | 28.640 | 876.302 | 0.756 | 13.1 | 2.867 | 7.081 |
| Archaea | 24.330 | 639.34 | 1.891 | 12.749 | 2.587 | 6.415 |
| Bacteria | 22.942 | 800.148 | 0.328 | 13.364 | 2.537 | 6.302 |
| Fungi | 98.911 | 703.133 | 3.396 | 11.959 | 3.478 | 6.964 |
| Human | 44.121 | 810.941 | 0.149 | 12.544 | 3.287 | 6.852 |
| Invertebrate | 44.585 | 1567.858 | 1.761 | 13.115 | 2.765 | 6.964 |
| Land Plants | 45.922 | 829.958 | 2.236 | 12.223 | 3.312 | 7.503 |
| Protozoa | 37.154 | 497.539 | 3.168 | 13.203 | 2.448 | 6.845 |
| Vertebrates | 43.493 | 925.221 | 0.525 | 13.217 | 2.511 | 7.088 |

Average molecular weight and isoelectric point of virus proteomes originated from different host. The virus protein of host fungi encoded the heaviest protein whereas virus protein of bacteria host encoded the lightest protein. However, virus proteome of bacteria host encoded highest *pI* (13.364) protein and protozoa host encoded lowest *pI* (2.448) protein.
