# Supplementary material for: Virtual 2D mapping of the viral proteome reveals host-specific modality distribution of molecular weight and isoelectric point
Source: Sci Rep. 2021 Oct 28;11:21291. doi: 10.1038/s41598-021-00797-3 (PMC8553790; doi:10.1038/s41598-021-00797-3)
Supplement: Supplementary file 8 — Supplementary Table 3. [file 41598_2021_797_MOESM8_ESM.docx]

| **Host** | **Average of Acidic *pI* proteins** | **Average of Basic *pI* proteins** |
| --- | --- | --- |
| Algae | 5.313 | 8.697 |
| Archaea | 5.001 | 8.696 |
| Bacteria | 5.236 | 8.130 |
| Fungi | 5.9 | 8.266 |
| Human | 5.531 | 8.231 |
| Invertebrate | 5.547 | 8.438 |
| Land Plants | 5.872 | 8.611 |
| Protozoa | 5.527 | 8.322 |
| Vertebrates | 5.635 | 8.559 |
| **Cumulative Average** | **5.507** | **8.439** |

**Supplementary Table 3.** Average of acidic and basic *pI* proteins in viral proteome with regard to their host. Highest basic *pI* protein was recorded in viruses associated with algae and archaea host whereas lowest basic *pI* protein was recorded in viruses associated with bacteria host. Similarly, highest acidic *pI* protein was recorded for viral proteome associated with fungal host and lowest acidic *pI* protein was recorded in viruses associated with archaea host.
